# 5-Cyclo­hexyl-3-(2-fluoro­phenyl­sulfon­yl)-2-methyl-1-benzo­furan

**DOI:** 10.1107/S1600536814005960

**Published:** 2014-03-22

**Authors:** Hong Dae Choi, Pil Ja Seo, Uk Lee

**Affiliations:** a Department of Chemistry, Dongeui University, San 24 Kaya-dong, Busanjin-gu, Busan 614-714, Republic of Korea; b Department of Chemistry, Pukyong National University, 599-1 Daeyeon 3-dong, Nam-gu, Busan 608-737, Republic of Korea

## Abstract

In the title compound, C_21_H_21_FO_3_S, the cyclo­hexyl ring adopts a chair conformation. The dihedral angle between the mean planes of the benzo­furan ring system and the fluoro­phenyl ring is 87.61 (3)°. In the crystal, mol­ecules related by inversion are linked into dimers *via* pairs of C—H⋯π inter­actions. These dimers are further linked by π–π inter­actions between the furan rings of neighbouring mol­ecules [centroid–centroid distance = 3.407 (2) Å and between the 2-fluoro­phenyl rings of neighbouring mol­ecules [centroid–centroid distance = 3.742 (2) Å], resulting in a three-dimensional supra­molecular network.

## Related literature   

For background information and the crystal structures of related compounds, see: Choi *et al.* (2012 ▶, 2014 ▶).
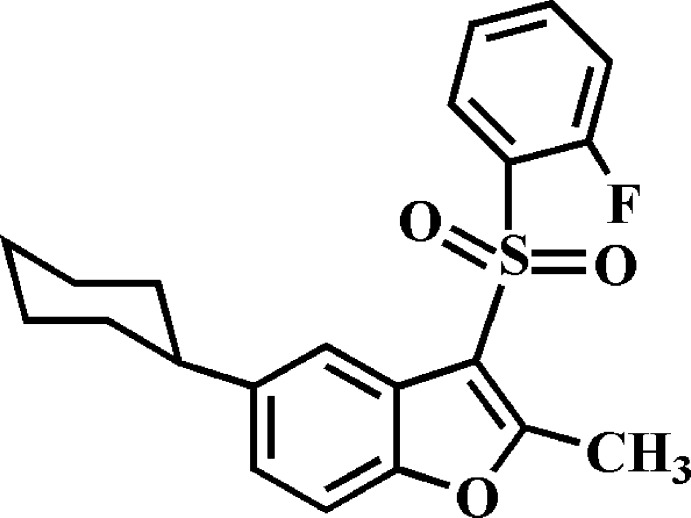


## Experimental   

### 

#### Crystal data  

C_21_H_21_FO_3_S
*M* *_r_* = 372.44
Triclinic, 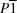

*a* = 9.0481 (2) Å
*b* = 10.5301 (2) Å
*c* = 10.6312 (2) Å
α = 106.025 (1)°
β = 92.561 (1)°
γ = 110.852 (1)°
*V* = 898.19 (3) Å^3^
*Z* = 2
Mo *K*α radiation
μ = 0.21 mm^−1^
*T* = 173 K
0.32 × 0.18 × 0.15 mm

#### Data collection  

Bruker SMART APEXII CCD diffractometer
Absorption correction: multi-scan (*SADABS*; Bruker, 2009 ▶) *T*_min_ = 0.683, *T*_max_ = 0.746
16579 measured reflections
4423 independent reflections
3920 reflections with *I* > 2σ(*I*)
*R*_int_ = 0.024

#### Refinement  

*R*[*F* ^2^ > 2σ(*F* ^2^)] = 0.040
*wR*(*F* ^2^) = 0.109
*S* = 1.04
4423 reflections
236 parameters
H-atom parameters constrained
Δρ_max_ = 0.38 e Å^−3^
Δρ_min_ = −0.37 e Å^−3^

### 

Data collection: *APEX2* (Bruker, 2009 ▶); cell refinement: *SAINT* (Bruker, 2009 ▶); data reduction: *SAINT*; program(s) used to solve structure: *SHELXS97* (Sheldrick, 2008 ▶); program(s) used to refine structure: *SHELXL97* (Sheldrick, 2008 ▶); molecular graphics: *ORTEP-3 for Windows* (Farrugia, 2012 ▶) and *DIAMOND* (Brandenburg, 1998 ▶); software used to prepare material for publication: *SHELXL97*.

## Supplementary Material

Crystal structure: contains datablock(s) I. DOI: 10.1107/S1600536814005960/zs2290sup1.cif

Structure factors: contains datablock(s) I. DOI: 10.1107/S1600536814005960/zs2290Isup2.hkl

Supporting information file. DOI: 10.1107/S1600536814005960/zs2290Isup3.cml

CCDC reference: 992238

Additional supporting information:  crystallographic information; 3D view; checkCIF report

## Figures and Tables

**Table 1:** Hydrogen-bond geometry (Å, °) *Cg*2 is the centroid of the C2–C7 benzene ring.

*D*—H⋯*A*	*D*—H	H⋯*A*	*D*⋯*A*	*D*—H⋯*A*
C14—H14*A*⋯*Cg*2^i^	0.99	2.73	3.644 (2)	154
C15—H15*B*⋯*Cg*2^ii^	0.98	2.80	3.454 (2)	125

Symmetry codes: (i) 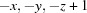; (ii) 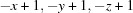.

**Fig. 1.**
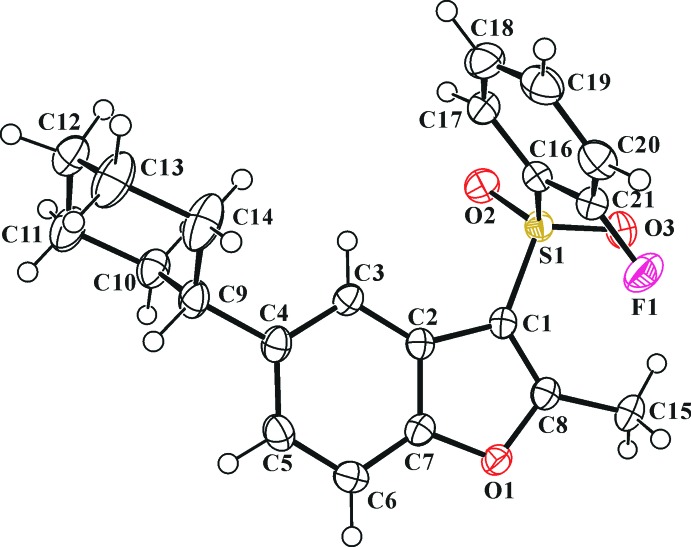
The molecular structure of the title molecule with the atom numbering scheme. The displacement ellipsoids are drawn at the 50% probability level. The hydrogen atoms are presented as small spheres of arbitrary radius.

**Fig. 2.**
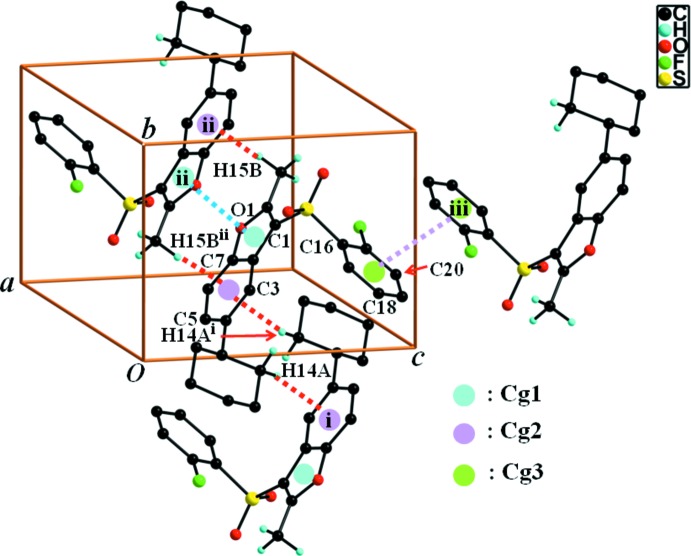
A view of the C—H···π and π–π interactions (dotted lines) in the crystal structure of the title compound. H atoms non-participating in hydrogen-bonding were omitted for clarity. [Symmetry codes: (i) - x, - y, - z + 1; (ii) - x + 1, - y + 1, - z + 1; (iii) - x, - y + 1, - z + 2.]

## supplementary crystallographic information

### 1. Comment

As a part of our ongoing study of 5-cyclohexyl-2-methyl-1-benzofuran derivatives containing 4-methylphenylsulfonyl (Choi *et al.*, 2012) and 2-bromophenylsulfonyl (Choi *et al.*, 2014) substituents in the 3-position, we report here on the crystal structure of the title compound.

In the title molecule (Fig. 1), the benzofuran ring system is essentially planar, with a mean deviation of 0.020 (1) Å from the least-squares plane defined by the nine constituent atoms. The 2-fluorophenyl ring is essentially planar, with a mean deviation of 0.007 (1) Å from the least-squares plane defined by the six constituent atoms. The cyclohexyl ring has a chair conformation. The dihedral angle formed by the benzofuran ring system and the 2-fluorophenyl ring is 87.61 (3)°.

In the crystal structure (Fig. 2), molecules related by inversion are paired into dimers via C—H···π interactions (Table 1, Cg2 is the centroid of the C2–C7 benzene ring). These dimers are further linked by π–π interactions; the first one between the furan rings of neighbouring molecules, with a Cg1···Cg1^ii^ distance of 3.407 (2) Å and an interplanar distance of 3.353 (2) Å resulting in a slippage of 0.604 (2) Å (Cg1 is the centroid of the C1/C2/C7/O1/C8 furan ring), and the second one between the 2-fluorophenyl rings of neighbouring molecules, with a Cg3···Cg3^iii^ distance of 3.742 (2) Å and an interplanar distance of 3.321 (2) Å resulting in a slippage of 1.724 (2) Å (Cg3 is the centroid of the C16–C21 2-fluorophenyl ring), forming a three-dimensional supramolecular network.

### 2. Experimental

3-Chloroperoxybenzoic acid (77%, 448 mg, 2.0 mmol) was added in small portions to a stirred solution of 5-cyclohexyl-3-(2-fluorophenylsulfanyl)-2-methyl-1-benzofuran (306 mg, 0.9 mmol) in dichloromethane (30 mL) at 273 K. After being stirred at room temperature for 8h, the mixture was washed with saturated sodium bicarbonate solution and the organic layer was separated, dried over magnesium sulfate, filtered and concentrated at reduced pressure. The residue was purified by column chromatography (benzene) to afford the title compound as a colorless solid [yield 73%, m.p. 437–438 K; *R*_f_ = 0.53 (benzene)]. Single crystals suitable for X-ray diffraction were prepared by slow evaporation of a solution of the title compound in benzene at room temperature.

### 3. Refinement

All H atoms were positioned geometrically and refined using a riding model, with C—H = 0.95 Å for aryl, 1.00 Å for methine, 0.99 Å for methylene and 0.98 Å for methyl H atoms, respectively. *U*_iso_ (H) = 1.2*U*_eq_ (C) for aryl, methine and methylene, and 1.5*U*_eq_ (C) for methyl H atoms. The positions of methyl hydrogens were optimized using the SHELXL-97's command AFIX 137 (Sheldrick, 2008).

### Crystal data

**Table:**

C_21_H_21_FO_3_S	*Z* = 2
*M**_r_* = 372.44	*F*(000) = 392
Triclinic, *P*1	*D*_x_ = 1.377 Mg m^−^^3^
Hall symbol: -P 1	Melting point = 437–438 K
*a* = 9.0481 (2) Å	Mo *K*α radiation, λ = 0.71073 Å
*b* = 10.5301 (2) Å	Cell parameters from 3275 reflections
*c* = 10.6312 (2) Å	θ = 2.6–28.0°
α = 106.025 (1)°	µ = 0.21 mm^−^^1^
β = 92.561 (1)°	*T* = 173 K
γ = 110.852 (1)°	Block, colourless
*V* = 898.19 (3) Å^3^	0.32 × 0.18 × 0.15 mm

### Data collection

**Table:**

Bruker SMART APEXII CCD diffractometer	4423 independent reflections
Radiation source: rotating anode	3920 reflections with *I* > 2σ(*I*)
Graphite multilayer monochromator	*R*_int_ = 0.024
Detector resolution: 10.0 pixels mm^-1^	θ_max_ = 28.3°, θ_min_ = 2.0°
φ and ω scans	*h* = −12→12
Absorption correction: multi-scan (*SADABS*; Bruker, 2009)	*k* = −14→12
*T*_min_ = 0.683, *T*_max_ = 0.746	*l* = −14→13
16579 measured reflections	

### Refinement

**Table:**

Refinement on *F*^2^	Primary atom site location: structure-invariant direct methods
Least-squares matrix: full	Secondary atom site location: difference Fourier map
*R*[*F*^2^ > 2σ(*F*^2^)] = 0.040	Hydrogen site location: difference Fourier map
*wR*(*F*^2^) = 0.109	H-atom parameters constrained
*S* = 1.04	*w* = 1/[σ^2^(*F*_o_^2^) + (0.0546*P*)^2^ + 0.3871*P*] where *P* = (*F*_o_^2^ + 2*F*_c_^2^)/3
4423 reflections	(Δ/σ)_max_ < 0.001
236 parameters	Δρ_max_ = 0.38 e Å^−^^3^
0 restraints	Δρ_min_ = −0.37 e Å^−^^3^

### Special details

**Table:**

Geometry. All esds (except the esd in the dihedral angle between two l.s. planes) are estimated using the full covariance matrix. The cell esds are taken into account individually in the estimation of esds in distances, angles and torsion angles; correlations between esds in cell parameters are only used when they are defined by crystal symmetry. An approximate (isotropic) treatment of cell esds is used for estimating esds involving l.s. planes.
Refinement. Refinement of F^2^ against ALL reflections. The weighted R-factor wR and goodness of fit S are based on F^2^, conventional R-factors R are based on F, with F set to zero for negative F^2^. The threshold expression of F^2^ > 2sigma(F^2^) is used only for calculating R-factors(gt) etc. and is not relevant to the choice of reflections for refinement. R-factors based on F^2^ are statistically about twice as large as those based on F, and R- factors based on ALL data will be even larger.

### Fractional atomic coordinates and isotropic or equivalent isotropic displacement parameters (Å^2^)

**Table:**

	*x*	*y*	*z*	*U*_iso_*/*U*_eq_	
S1	0.23801 (4)	0.57051 (4)	0.70681 (3)	0.02569 (11)	
F1	0.28674 (11)	0.45682 (12)	0.92462 (9)	0.0410 (2)	
O1	0.53546 (11)	0.38158 (11)	0.59971 (10)	0.0263 (2)	
O2	0.15677 (13)	0.59421 (12)	0.60205 (11)	0.0343 (2)	
O3	0.34140 (14)	0.69153 (11)	0.81440 (11)	0.0354 (3)	
C1	0.34106 (16)	0.46540 (14)	0.63767 (13)	0.0233 (3)	
C2	0.27505 (16)	0.33440 (14)	0.52683 (13)	0.0225 (3)	
C3	0.12613 (16)	0.25272 (14)	0.44701 (14)	0.0254 (3)	
H3	0.0394	0.2835	0.4575	0.030*	
C4	0.10766 (17)	0.12480 (15)	0.35152 (14)	0.0276 (3)	
C5	0.23950 (19)	0.08429 (16)	0.33530 (15)	0.0318 (3)	
H5	0.2257	−0.0022	0.2686	0.038*	
C6	0.38875 (18)	0.16516 (16)	0.41251 (15)	0.0309 (3)	
H6	0.4774	0.1373	0.4000	0.037*	
C7	0.40081 (16)	0.28817 (15)	0.50839 (14)	0.0248 (3)	
C8	0.49546 (16)	0.48773 (15)	0.67799 (13)	0.0247 (3)	
C9	−0.05240 (18)	0.02861 (15)	0.26472 (16)	0.0320 (3)	
H9	−0.0466	−0.0665	0.2220	0.038*	
C10	−0.08901 (18)	0.08408 (18)	0.15404 (15)	0.0346 (3)	
H10A	−0.0001	0.0985	0.1017	0.042*	
H10B	−0.0975	0.1777	0.1930	0.042*	
C11	−0.2454 (2)	−0.0209 (2)	0.06288 (17)	0.0457 (4)	
H11A	−0.2689	0.0201	−0.0052	0.055*	
H11B	−0.2325	−0.1110	0.0167	0.055*	
C12	−0.38452 (19)	−0.05379 (19)	0.13816 (18)	0.0398 (4)	
H12A	−0.4088	0.0329	0.1722	0.048*	
H12B	−0.4803	−0.1295	0.0771	0.048*	
C13	−0.3493 (2)	−0.1026 (2)	0.2526 (2)	0.0520 (5)	
H13A	−0.3422	−0.1974	0.2178	0.062*	
H13B	−0.4383	−0.1133	0.3047	0.062*	
C14	−0.1923 (2)	0.0033 (2)	0.34305 (19)	0.0509 (5)	
H14A	−0.1703	−0.0346	0.4141	0.061*	
H14B	−0.2030	0.0954	0.3852	0.061*	
C15	0.62304 (18)	0.59702 (17)	0.78620 (15)	0.0318 (3)	
H15A	0.5870	0.6729	0.8309	0.048*	
H15B	0.7197	0.6380	0.7494	0.048*	
H15C	0.6469	0.5526	0.8501	0.048*	
C16	0.08913 (16)	0.45768 (15)	0.77301 (14)	0.0255 (3)	
C17	−0.07163 (18)	0.41291 (16)	0.72256 (15)	0.0315 (3)	
H17	−0.1022	0.4380	0.6491	0.038*	
C18	−0.18705 (19)	0.33113 (17)	0.78072 (18)	0.0376 (4)	
H18	−0.2973	0.3007	0.7473	0.045*	
C19	−0.1428 (2)	0.29361 (17)	0.88695 (17)	0.0374 (4)	
H19	−0.2231	0.2390	0.9268	0.045*	
C20	0.0174 (2)	0.33479 (17)	0.93587 (16)	0.0342 (3)	
H20	0.0482	0.3075	1.0077	0.041*	
C21	0.13039 (17)	0.41607 (16)	0.87779 (14)	0.0293 (3)	

### Atomic displacement parameters (Å^2^)

**Table:**

	*U*^11^	*U*^22^	*U*^33^	*U*^12^	*U*^13^	*U*^23^
S1	0.02763 (18)	0.02397 (18)	0.02401 (18)	0.01045 (14)	0.00101 (13)	0.00503 (13)
F1	0.0303 (5)	0.0630 (6)	0.0340 (5)	0.0184 (5)	0.0005 (4)	0.0216 (5)
O1	0.0215 (5)	0.0309 (5)	0.0253 (5)	0.0089 (4)	0.0023 (4)	0.0084 (4)
O2	0.0394 (6)	0.0362 (6)	0.0336 (6)	0.0197 (5)	0.0025 (5)	0.0142 (5)
O3	0.0368 (6)	0.0255 (5)	0.0337 (6)	0.0086 (4)	−0.0001 (5)	−0.0009 (4)
C1	0.0234 (6)	0.0234 (6)	0.0205 (6)	0.0065 (5)	0.0022 (5)	0.0065 (5)
C2	0.0239 (6)	0.0223 (6)	0.0205 (6)	0.0071 (5)	0.0038 (5)	0.0079 (5)
C3	0.0231 (6)	0.0238 (6)	0.0273 (7)	0.0070 (5)	0.0009 (5)	0.0082 (5)
C4	0.0290 (7)	0.0223 (6)	0.0272 (7)	0.0056 (5)	−0.0003 (5)	0.0077 (5)
C5	0.0374 (8)	0.0248 (7)	0.0292 (7)	0.0118 (6)	0.0033 (6)	0.0031 (6)
C6	0.0302 (7)	0.0318 (7)	0.0327 (8)	0.0150 (6)	0.0070 (6)	0.0086 (6)
C7	0.0219 (6)	0.0273 (7)	0.0238 (6)	0.0065 (5)	0.0034 (5)	0.0100 (5)
C8	0.0251 (6)	0.0263 (6)	0.0221 (6)	0.0075 (5)	0.0033 (5)	0.0100 (5)
C9	0.0326 (7)	0.0203 (6)	0.0352 (8)	0.0059 (6)	−0.0056 (6)	0.0040 (6)
C10	0.0299 (7)	0.0409 (8)	0.0268 (7)	0.0060 (6)	0.0033 (6)	0.0112 (6)
C11	0.0358 (9)	0.0595 (11)	0.0302 (8)	0.0076 (8)	−0.0027 (7)	0.0117 (8)
C12	0.0289 (8)	0.0385 (9)	0.0431 (9)	0.0060 (7)	−0.0025 (7)	0.0096 (7)
C13	0.0365 (9)	0.0476 (10)	0.0545 (11)	−0.0088 (8)	−0.0055 (8)	0.0247 (9)
C14	0.0373 (9)	0.0574 (11)	0.0387 (9)	−0.0108 (8)	−0.0028 (7)	0.0252 (9)
C15	0.0266 (7)	0.0334 (8)	0.0284 (7)	0.0063 (6)	−0.0041 (6)	0.0074 (6)
C16	0.0262 (7)	0.0264 (6)	0.0226 (6)	0.0117 (5)	0.0032 (5)	0.0039 (5)
C17	0.0289 (7)	0.0324 (7)	0.0306 (7)	0.0136 (6)	−0.0001 (6)	0.0043 (6)
C18	0.0265 (7)	0.0330 (8)	0.0466 (9)	0.0093 (6)	0.0032 (7)	0.0055 (7)
C19	0.0366 (8)	0.0283 (7)	0.0439 (9)	0.0107 (6)	0.0135 (7)	0.0076 (7)
C20	0.0406 (8)	0.0345 (8)	0.0306 (8)	0.0172 (7)	0.0098 (6)	0.0108 (6)
C21	0.0279 (7)	0.0337 (7)	0.0252 (7)	0.0138 (6)	0.0017 (5)	0.0054 (6)

### Geometric parameters (Å, º)

**Table:**

S1—O2	1.4331 (11)	C10—H10B	0.9900
S1—O3	1.4347 (11)	C11—C12	1.509 (2)
S1—C1	1.7267 (14)	C11—H11A	0.9900
S1—C16	1.7688 (15)	C11—H11B	0.9900
F1—C21	1.3492 (17)	C12—C13	1.508 (3)
O1—C8	1.3690 (17)	C12—H12A	0.9900
O1—C7	1.3814 (16)	C12—H12B	0.9900
C1—C8	1.3593 (19)	C13—C14	1.531 (2)
C1—C2	1.4495 (18)	C13—H13A	0.9900
C2—C7	1.3900 (19)	C13—H13B	0.9900
C2—C3	1.3948 (18)	C14—H14A	0.9900
C3—C4	1.3938 (19)	C14—H14B	0.9900
C3—H3	0.9500	C15—H15A	0.9800
C4—C5	1.405 (2)	C15—H15B	0.9800
C4—C9	1.5149 (19)	C15—H15C	0.9800
C5—C6	1.385 (2)	C16—C21	1.386 (2)
C5—H5	0.9500	C16—C17	1.389 (2)
C6—C7	1.374 (2)	C17—C18	1.388 (2)
C6—H6	0.9500	C17—H17	0.9500
C8—C15	1.4781 (19)	C18—C19	1.382 (2)
C9—C10	1.523 (2)	C18—H18	0.9500
C9—C14	1.531 (2)	C19—C20	1.386 (2)
C9—H9	1.0000	C19—H19	0.9500
C10—C11	1.528 (2)	C20—C21	1.373 (2)
C10—H10A	0.9900	C20—H20	0.9500
			
O2—S1—O3	118.98 (7)	C12—C11—H11B	109.2
O2—S1—C1	108.44 (7)	C10—C11—H11B	109.2
O3—S1—C1	109.88 (7)	H11A—C11—H11B	107.9
O2—S1—C16	106.95 (7)	C13—C12—C11	111.81 (15)
O3—S1—C16	108.27 (7)	C13—C12—H12A	109.3
C1—S1—C16	103.14 (6)	C11—C12—H12A	109.3
C8—O1—C7	107.02 (10)	C13—C12—H12B	109.3
C8—C1—C2	107.83 (12)	C11—C12—H12B	109.3
C8—C1—S1	126.81 (11)	H12A—C12—H12B	107.9
C2—C1—S1	125.35 (10)	C12—C13—C14	111.71 (14)
C7—C2—C3	119.77 (13)	C12—C13—H13A	109.3
C7—C2—C1	104.36 (12)	C14—C13—H13A	109.3
C3—C2—C1	135.83 (13)	C12—C13—H13B	109.3
C4—C3—C2	118.38 (13)	C14—C13—H13B	109.3
C4—C3—H3	120.8	H13A—C13—H13B	107.9
C2—C3—H3	120.8	C13—C14—C9	111.01 (16)
C3—C4—C5	119.56 (13)	C13—C14—H14A	109.4
C3—C4—C9	121.05 (13)	C9—C14—H14A	109.4
C5—C4—C9	119.40 (13)	C13—C14—H14B	109.4
C6—C5—C4	122.76 (14)	C9—C14—H14B	109.4
C6—C5—H5	118.6	H14A—C14—H14B	108.0
C4—C5—H5	118.6	C8—C15—H15A	109.5
C7—C6—C5	115.93 (14)	C8—C15—H15B	109.5
C7—C6—H6	122.0	H15A—C15—H15B	109.5
C5—C6—H6	122.0	C8—C15—H15C	109.5
C6—C7—O1	125.87 (13)	H15A—C15—H15C	109.5
C6—C7—C2	123.55 (13)	H15B—C15—H15C	109.5
O1—C7—C2	110.57 (12)	C21—C16—C17	119.12 (14)
C1—C8—O1	110.21 (12)	C21—C16—S1	120.80 (11)
C1—C8—C15	134.27 (13)	C17—C16—S1	120.05 (11)
O1—C8—C15	115.50 (12)	C18—C17—C16	119.21 (15)
C4—C9—C10	112.58 (12)	C18—C17—H17	120.4
C4—C9—C14	113.05 (13)	C16—C17—H17	120.4
C10—C9—C14	109.00 (14)	C19—C18—C17	120.51 (15)
C4—C9—H9	107.3	C19—C18—H18	119.7
C10—C9—H9	107.3	C17—C18—H18	119.7
C14—C9—H9	107.3	C18—C19—C20	120.68 (15)
C9—C10—C11	111.03 (13)	C18—C19—H19	119.7
C9—C10—H10A	109.4	C20—C19—H19	119.7
C11—C10—H10A	109.4	C21—C20—C19	118.24 (15)
C9—C10—H10B	109.4	C21—C20—H20	120.9
C11—C10—H10B	109.4	C19—C20—H20	120.9
H10A—C10—H10B	108.0	F1—C21—C20	118.76 (14)
C12—C11—C10	112.06 (14)	F1—C21—C16	119.04 (13)
C12—C11—H11A	109.2	C20—C21—C16	122.20 (14)
C10—C11—H11A	109.2		
			
O2—S1—C1—C8	133.20 (13)	C7—O1—C8—C15	177.95 (11)
O3—S1—C1—C8	1.62 (15)	C3—C4—C9—C10	76.96 (18)
C16—S1—C1—C8	−113.64 (13)	C5—C4—C9—C10	−103.00 (16)
O2—S1—C1—C2	−48.36 (13)	C3—C4—C9—C14	−47.1 (2)
O3—S1—C1—C2	−179.93 (11)	C5—C4—C9—C14	132.94 (16)
C16—S1—C1—C2	64.81 (12)	C4—C9—C10—C11	175.95 (14)
C8—C1—C2—C7	−0.63 (14)	C14—C9—C10—C11	−57.79 (18)
S1—C1—C2—C7	−179.32 (10)	C9—C10—C11—C12	56.0 (2)
C8—C1—C2—C3	177.13 (15)	C10—C11—C12—C13	−53.0 (2)
S1—C1—C2—C3	−1.6 (2)	C11—C12—C13—C14	53.1 (2)
C7—C2—C3—C4	0.93 (19)	C12—C13—C14—C9	−56.2 (2)
C1—C2—C3—C4	−176.57 (14)	C4—C9—C14—C13	−176.00 (15)
C2—C3—C4—C5	−2.1 (2)	C10—C9—C14—C13	58.0 (2)
C2—C3—C4—C9	177.92 (13)	O2—S1—C16—C21	−179.39 (11)
C3—C4—C5—C6	1.3 (2)	O3—S1—C16—C21	−50.05 (13)
C9—C4—C5—C6	−178.75 (14)	C1—S1—C16—C21	66.36 (13)
C4—C5—C6—C7	0.8 (2)	O2—S1—C16—C17	−1.21 (14)
C5—C6—C7—O1	177.24 (13)	O3—S1—C16—C17	128.13 (12)
C5—C6—C7—C2	−2.0 (2)	C1—S1—C16—C17	−115.46 (12)
C8—O1—C7—C6	−178.82 (13)	C21—C16—C17—C18	1.8 (2)
C8—O1—C7—C2	0.53 (14)	S1—C16—C17—C18	−176.37 (11)
C3—C2—C7—C6	1.2 (2)	C16—C17—C18—C19	−0.5 (2)
C1—C2—C7—C6	179.43 (13)	C17—C18—C19—C20	−1.1 (2)
C3—C2—C7—O1	−178.14 (11)	C18—C19—C20—C21	1.3 (2)
C1—C2—C7—O1	0.06 (14)	C19—C20—C21—F1	179.99 (13)
C2—C1—C8—O1	0.99 (15)	C19—C20—C21—C16	0.1 (2)
S1—C1—C8—O1	179.66 (9)	C17—C16—C21—F1	178.43 (13)
C2—C1—C8—C15	−177.62 (14)	S1—C16—C21—F1	−3.38 (19)
S1—C1—C8—C15	1.0 (2)	C17—C16—C21—C20	−1.7 (2)
C7—O1—C8—C1	−0.95 (15)	S1—C16—C21—C20	176.52 (12)

### Hydrogen-bond geometry (Å, º)

Cg2 is the centroid of the C2–C7 benzene ring.

**Table:**

*D*—H···*A*	*D*—H	H···*A*	*D*···*A*	*D*—H···*A*
C14—H14*A*···*Cg*2^i^	0.99	2.73	3.644 (2)	154
C15—H15*B*···*Cg*2^ii^	0.98	2.80	3.454 (2)	125

Symmetry codes: (i) −*x*, −*y*, −*z*+1; (ii) −*x*+1, −*y*+1, −*z*+1.
